# Glances at the Hospitals

**Published:** 1900-11-10

**Authors:** 


### GLANCES AT THE HOSPITALS.

### THE HASTINGS AND EAST SUSSEX HOSPITAL.

The receipts during 1899 amounted to £3,752, and the expenditure to £4,271; the former are rather below the average for the previous three years and the latter is about £200 above the average. The surplus of assets over liabilities is £13,046, and it should be pointed out that although there is a deficiency in the receipts, it is less than it was in 1898, and the Committee appeal to those who have not yet become annual subscribers to help them now. The subscriptions for the year were £1,501; but it does not appear whether workmen's subscriptions are or are not included in that sum. If not, it would be possible to raise a considerable amount annually from "workmen's subscriptions" or from patients' payments. During 1899 the St. Helens Hospital received nearly £1,700 from these sources. On January 1st the Hastings Hospital contained 52 patients and 685 were admitted during the year. Of these 466 were discharged recovered, 122 relieved, 50 for other reasons, 2 were brought in dead, 14 died within 48 hours, 37 others died, and 46 remained under treatment. The out-patients numbered 1,090, and of these 956 were cured; 96 were made in-patients. The medical and surgical tables convey no information beyond naming the diseases treated and the number of patients suffering from these diseases, but of the results we hear nothing. Similar remarks may be made of the operations. It is stated that 464 were performed. Among them were 13 abdominal sections, and various others of which we should have liked to hear the terminations. These tables require to be completely remodelled in the next report. The financial report should also contain a clear statement of the cost per bed occupied, of the cost per patient, and of the cost per out-patient.

### THE MANCHESTER HOSPITAL FOR CONSUMPTION AND THROAT DISEASES.

The total cost of in-patients' department was £3,535, but his included a sum of £1,279 spent on the verandahs, in which the open-air treatment of phthisis is being carried out—it is hoped with good results. The total cost per week of each patient was 17s. 1½d., and the average payments made by patients have increased from 1s. 1½d. to 2s. 2d. a week. The committee think this is a very satisfactory sign, as it shows "a desire on the part of the patients to help themselves;" and we must agree with the committee when we learn that the total sum received from patients during the year approached £1,000. We are also told that the voluntary notification of phthisis has been introduced in Manchester; and that in a very few cases only has any objection been raised to the practical working of the scheme. A new hospital is to be built at Delamere. On January 1st, 1899, the hospital contained fifty-one patients, and 280 were admitted during the year. Of these, 275 were discharged and 49 were under treatment on December 31st, 1899. There were 145 cases of undoubted phthisis, and of these 47 were much improved; 52 improved, 25 were stationary, 14 were worse, and 7 died. Looking at the weights of the 138 cases, we find that 113 gained an average of 8 lb.; 21 lost an average of 5 lb., and 4 neither lost nor gained. As to the innovation of the outdoor treatment, the medical officers give a very sensible summing up; and they think the experiment has been "distinctly encouraging," especially when the site of the hospital is taken into account; and "it is therefore with good hope of achieving even better results that we look forward to the carrying out of our Chairman's great scheme of building a hospital on a site sufficiently remote from any considerable centre of population to ensure really pure air for our patients to breathe."

### ST. HELEN'S HOSPITAL.

The ordinary income for the year amounted to £2,004 and the ordinary expenditure to £1,797, thus leaving a credit balance of upwards of £200, which sum was further increased by a legacy of £1,000. Law expenses and the payment of money spent on the enlargement of the hospital have reduced this balance to a little further than the vanishing-point. The information supplied to us by the medical tables is insufficient. We learn, however, that 32 patients were in the hospital at the beginning of the year, and 34 at the end of it; that 347 were admitted during the twelve months; that 252 were discharged cured, 32 relieved, 11 not relieved, 8 for various reasons, 7 to the Southport Convalescent Hospital, 4 to Buxton "Devonshire" Hospital, and that 31 died. We trust the next annual report will give some details of the results of treatment, and a table showing the operations performed.

